# Supplementary material for: The Mars1 kinase confers photoprotection through signaling in the chloroplast unfolded protein response
Source: eLife. 2019 Oct 15;8:e49577. doi: 10.7554/eLife.49577 (PMC6794094; doi:10.7554/eLife.49577)
Supplement: Source code 1. [file elife-49577-code1.docx]

*Macro #1 separates individual 384-well plates from an arrayed image, using plates imaged in the chlorophyll channel as a template to crop and save the YFP (UNSEP1) and chlorophyll (UNSEP2) image for each 384-well plate.*

unsep2 = File.openDialog("Choose the original UNSEP2 file");

unsep1= File.openDialog("Choose the original UNSEP1 file");

setBatchMode(true);

dir2=getDirectory("Make a new directory for UNSEP2");

dir1=getDirectory("Make a new directory for UNSEP1");

open(unsep2);

plate = getNumber("How many plates are there?", 0);

setThreshold(266, 37000);

run("Set Measurements...", " center limit display redirect=None decimal=3");

run("Analyze Particles...", "size=0.002-Infinity circularity=0.70-1.00 display");

cornerX=getResult("XM",0);

cornerY=getResult("YM",0);

for(j=1;j<nResults;j++){

xnew = getResult("XM",j);

ynew = getResult("YM",j);

if (cornerX > xnew){

cornerX = xnew;

}

if (cornerY > ynew){

cornerY = ynew;

}

}

run("ROI Manager...");

width = 1260;

height = 840;

x = (100*cornerX)-width/12;

y = (100*cornerY)-height/8;

spacing = 30;

numRow = 4;

numCol = plate/numRow;

for(b = 0; b < numRow; b++)

{

for(d = 0; d < numCol; d++)

{

xOffset = d * (width + spacing);

yOffset = b * (height + spacing);

makeRectangle(x + xOffset, y + yOffset, width, height);

roiManager("Add");

}

}

n = roiManager("count");

for (j=0;j<n;j++){

open(unsep1);

roiManager("Select", j);

plate = getString("Enter plate number","100");

run("Duplicate...","title=placeholder");

saveAs("Tiff", dir1+plate+"-"+"UNSEP1-"+".tif");

close();

close();

open(unsep2);

roiManager("Select", j);

run("Duplicate...","title=placeholder");

saveAs("Tiff", dir2+plate+"-"+"UNSEP2-"+".tif");

close();

close();

}

selectWindow("ROI Manager");

run("Close");

close();

*Macro #2a takes the individual plate images saved from Macro #1 and allows the user to re-orient the 384-well plate by eye, looking for the specific YFP fluorescence pattern conferred by the positive and negative reporter strains in the bottom two rows.*

dirUNSEP2=getDirectory("Choose Directory of Individual UNSEP2 Plates");

//choose directory with images cropped into individual plates containing UNSEP2 or chlorophyll channel

dirUNSEP1=getDirectory("Choose Directory of Individual UNSEP1 Plates");

//choose directory with images cropped into individual plates containing YFP channel

listUNSEP1=getFileList(dirUNSEP1);

listUNSEP2=getFileList(dirUNSEP2);

//command gets a list of the files in the chosen directories

for (i=0; i<listUNSEP1.length; i++) {

showProgress(i+1, listUNSEP1.length);

open(dirUNSEP1+listUNSEP1[i]);

n = getBoolean("P1 at bottom left corner?");

if(n==0){

run("Flip Horizontally");

run("Save", dirUNSEP1+listUNSEP1[i]);

run("Close");

open(dirUNSEP2+listUNSEP2[i]);

run("Flip Horizontally");

run("Save", dirUNSEP2+listUNSEP2[i]);

close();

i = i-1;

}

else{

run("Close");

}

}

*Macro #2b takes the oriented chlorophyll (UNSEP2) image of each plate and builds a 16x24 grid based on the detection of a chlorophyll-positive colony in the top leftmost corner as A1. This grid is saved and applied to the YFP (UNSEP1) image in subsequent scripts.*

dir1=getDirectory("Choose Directory of Cropped UNSEP2 Plates");

//choose directory with images cropped into individual plates containing UNSEP2 or chlorophyll channel

dir2=getDirectory("Create Output Directory for UNSEP2 ROIs");

list=getFileList(dir1);

//command gets a list of the files in the chosen directories

setBatchMode(true);

for (m=0; m<list.length; m++) {

showProgress(m+1, list.length);

open(dir1+list[m]);

setThreshold(266, 37000);

run("Set Measurements...", " center limit display redirect=None decimal=3");

run("Analyze Particles...", "size=0.002-Infinity circularity=0.70-1.00 display");

cornerX=getResult("XM",0);

cornerY=getResult("YM",0);

for(j=1;j<nResults;j++){

xnew = getResult("XM",j);

ynew = getResult("YM",j);

if (cornerX > xnew){

cornerX = xnew;

}

if (cornerY > ynew){

cornerY = ynew;

}

}

alphabet = newArray("A","B","C","D","E","F","G","H","I","J","K","L","M","N","O","P");

run("ROI Manager...");

width = 42;

height = 42;

x = (100*cornerX)-width/2;

y = (100*cornerY)-height/2;

spacing = 3;

numRow = 16;

numCol = 24;

for(b = 0; b < numRow; b++)

{

for(d = 0; d < numCol; d++)

{

xOffset = d * (width + spacing);

yOffset = b * (height + spacing);

makeRectangle(x + xOffset, y + yOffset, width, height);

roiManager("Add");

}

}

k=1;

for(j=0;j<alphabet.length;j++){

for (i=1; i<25; i++) {

roiManager("select",k-1);

roiManager("rename",(k)+": "+alphabet[j]+"-"+(i));

k=k+1;

}

}

run("Select All");

roiManager("Save", dir2+list[m]+"-ROI.zip");

selectWindow("ROI Manager");

run("Close");

run("Clear Results");

selectWindow("Results");

run("Close");

close();

}

*Macro #3 allows the user to iterate through images of the individual 384-well plates and their assigned 16x24 grids to check that the grid is properly centered on all colonies. If the grid is misaligned, users have the opportunity to input a new position for the A1 (top leftmost) corner.*

dirUNSEP2=getDirectory("Choose Directory of Individual UNSEP2 Plates");

//choose directory with images cropped into individual plates containing UNSEP2 or chlorophyll channel

dirROI=getDirectory("Choose Directory with the ROIs");

//choose directory with ROI grids built for the UNSEP2 channel

listUNSEP2=getFileList(dirUNSEP2);

listROI=getFileList(dirROI);

Array.sort(listUNSEP2);

Array.sort(listROI);

//command gets a list of the files in the chosen directories

alphabet = newArray("A","B","C","D","E","F","G","H","I","J","K","L","M","N","O","P");

for (i=0; i<listUNSEP2.length; i++) {

showProgress(i+1, listUNSEP2.length);

open(dirUNSEP2+listUNSEP2[i]);

run("ROI Manager...");

open(dirROI+listROI[i]);

roiManager("Show All");

n = getBoolean("Does the grid fit?");

if(n==0){

selectWindow("ROI Manager");

run("Close");

File.delete(dirROI+listROI[i]);

run("ROI Manager...");

cornerX = getNumber("X value?", 0);

cornerY = getNumber("Y value?", 0);

width = 42;

height = 42;

x = (100*cornerX)-width/2;

y = (100*cornerY)-height/2;

spacing = 3;

numRow = 16;

numCol = 24;

for(b = 0; b < numRow; b++)

{

for(d = 0; d < numCol; d++)

{

xOffset = d * (width + spacing);

yOffset = b * (height + spacing);

makeRectangle(x + xOffset, y + yOffset, width, height);

roiManager("Add");

}

}

k=1;

for(j=0;j<alphabet.length;j++){

for (p=1; p<25; p++) {

roiManager("select",k-1);

roiManager("rename",(k)+": "+alphabet[j]+"-"+(p));

k=k+1;

}

}

run("Select All");

roiManager("Save", dirROI+listUNSEP2[i]+"-ROI.zip");

selectWindow("ROI Manager");

run("Close");

run("Clear Results");

close();

i = i-1;

}

else{

selectWindow("ROI Manager");

run("Close");

selectWindow(listUNSEP2[i]);

close();

}

}

*Macro #4a overlays the assigned 16x24 grids onto their respective images in both the YFP and chlorophyll image and measures the maximum intensity within each grid region.*

dirYFP=getDirectory("Choose Directory with Individual UNSEP1 Plates");

//choose directory with images cropped into individual plates containing UNSEP1 or YFP channel

dirCphyll=getDirectory("Choose Directory with Individual UNSEP2 Plates");

//choose directory with images cropped into individual plates containing UNSEP2 or chlorophyll channel

dirROI=getDirectory("Choose Directory of ROIs");

//choose directory with ROIs made from the Chlorophyll channel

dir3=getDirectory("Choose Output Directory for YFP");

dir4=getDirectory("Choose Output Directory for chlorophyll");

//choose directory where the excel files go

list1=getFileList(dirYFP);

list2=getFileList(dirCphyll);

list3=getFileList(dirROI);

Array.sort(list1);

Array.sort(list2);

Array.sort(list3);

setBatchMode(true);

run("Set Measurements...", "area mean standard min integrated display redirect=None decimal=3");

run("ROI Manager...");

for (i=0; i<list1.length; i++) {

showProgress(i+1, list1.length);

open(dirYFP+list1[i]);

run("ROI Manager...");

open(dirROI+list3[i]);

rcount = roiManager("count");

for (j=0;j<rcount;j++){

roiManager("Select", j);

roiManager("Measure");

}

saveAs("Results", dir3+list1[i]+"-YFP Intensities.xls");

run("Clear Results");

close();

selectWindow("ROI Manager");

run("Close");

}

for (i=0; i<list2.length; i++) {

showProgress(i+1, list2.length);

run("Set Measurements...", "area mean standard min integrated display redirect=None decimal=3");

open(dirCphyll+list2[i]);

run("ROI Manager...");

open(dirROI+list3[i]);

rcount = roiManager("count");

for (j=0;j<rcount-1;j++){

roiManager("Select", j);

roiManager("Measure");

}

saveAs("Results", dir4+list2[i]+"-Chlorophyll Intensities.xls");

run("Clear Results");

selectWindow("Results");

run("Close");

selectWindow("ROI Manager");

run("Close");

}

close();

*Macro #4b overlays the assigned 16x24 grids onto their respective images in the chlorophyll image and measures the area of the region of intensity above a specified threshold within each grid region, representing the area of the colony.*

dirCphyll=getDirectory("Choose Directory with Individual UNSEP2 Plates");

//choose directory with images cropped into individual plates containing UNSEP2 or chlorophyll channel

dirROI=getDirectory("Choose Directory of ROIs");

//choose directory with ROIs made from the Chlorophyll channel

dir4=getDirectory("Choose Output Directory for chlorophyll area");

//choose directory where the excel files go

list2=getFileList(dirCphyll);

list3=getFileList(dirROI);

Array.sort(list2);

Array.sort(list3);

setBatchMode(true);

run("Set Measurements...", "area limit display redirect=None decimal=3");

run("ROI Manager...");

for (i=0; i<list2.length; i++) {

showProgress(i+1, list2.length);

open(dirCphyll+list2[i]);

setThreshold(300, 37000);

run("ROI Manager...");

open(dirROI+list3[i]);

rcount = roiManager("count");

for (j=0;j<rcount-1;j++){

roiManager("Select", j);

roiManager("Measure");

}

saveAs("Results", dir4+list2[i]+"- Chlorophyll Areas.xls");

run("Clear Results");

selectWindow("Results");

run("Close");

selectWindow("ROI Manager");

run("Close");

}

close();

*Macro #5a collects the YFP intensities measured from Macro #4a and identifies the parental colonies associated with each plate (positions 339, 340, 347, 348, 351, 352, 363, 364, 371, 372, 375, and 376) to find the average measured intensity of these parental colonies with and without vitamin. The average measured intensity is then used to normalize the measured intensity for each candidate colony.*

dirYFP=getDirectory("Choose Directory with YFP Excel Sheets");

dir2=getDirectory("Choose output file for YFP max plus/no Vit ratios");

list1=getFileList(dirYFP);

Array.sort(list1);

setBatchMode(true);

for (i=0; i<list1.length; i+=2) {

showProgress(i+1, list1.length);

open(dirYFP+list1[i]);

YFPnoVit = newArray(nResults);

YFPplusVit = newArray(nResults);

YLabel = newArray(nResults);

YFPratio= newArray(nResults);

normYFPratio= newArray(nResults);

lognormYFPratio= newArray(nResults);

controlA1N5 = newArray(339,340,347,348,351,352,363,364,371,372,375,376);

contotal = 0;

for (j=0;j<nResults;j++){

YFPnoVit[j]=getResult("Max",j);

YLabel[j]= getResultLabel(j);

}

selectWindow("Results");

run("Close");

open(dirYFP+list1[i+1]);

for (j=0;j<nResults;j++){

YFPplusVit[j]=getResult("Max",j);

}

selectWindow("Results");

run("Close");

for (j=0;j<YFPplusVit.length;j++){

Yplus=YFPplusVit[j];

Yno=YFPnoVit[j];

YFPratio[j]=Yplus/Yno;

}

for (j=0;j<controlA1N5.length;j++){

pos = controlA1N5[j]-1;

contotal = contotal + YFPratio[pos];

}

conmean=contotal/controlA1N5.length;

for (j=0;j<YFPratio.length;j++){

normYFPratio[j]=YFPratio[j]/conmean;

lognormYFPratio[j]=log(normYFPratio[j])/log(10);

}

for (m=0; m<YFPratio.length; m++) {

setResult("Label",m,YLabel[m]);

setResult("Max: plus to no Vit YFP Ratio",m,YFPratio[m]);

setResult("norm Max: plus to no Vit YFP Ratio",m,normYFPratio[m]);

setResult("log norm-Max: plus to no Vit YFP Ratio",m,lognormYFPratio[m]);

}

saveAs("Results", dir2+YLabel[0]+" YFPmax-plustonoVit-Ratio.xls");

}

close();

*Macro #5b takes the colony areas obtained from Macro #4b for chlorophyll images obtained after 2 days and 6 days of vitamin treatment to find a ratio for each candidate colony that reflects the growth of the colony upon vitamin treatment.*

dirArea48hrs=getDirectory("Choose Directory with Area at 48 hrs Excel Sheets");

dirArea6days=getDirectory("Choose Directory with Area at 6 days Excel Sheets");

dir2=getDirectory("Choose output file for Area ratios");

list48=getFileList(dirArea48hrs);

list6d=getFileList(dirArea6days);

Array.sort(list48);

Array.sort(list6d);

setBatchMode(true);

mutstart = 0;

mutend = 336;

muttotal = mutend-mutstart;

mutLabelArea = newArray(muttotal*list48.length/2);

normArearat = newArray(muttotal*list48.length/2);

for (i=0; i<list6d.length; i++) {

showProgress(i+1, list6d.length);

open(dirArea6days+list6d[i]);

Area48hrs = newArray(nResults);

Area6days = newArray(nResults);

AreaLabel = newArray(nResults);

Arearatio= newArray(nResults);

Arealogratio= newArray(nResults);

normArealogratio= newArray(nResults);

controlA1N5 = newArray(339,340,347,348,351,352,363,364,371,372,375,376);

totallog = 0;

for (j=0;j<nResults;j++){

Area6days[j]=getResult("Area",j);

AreaLabel[j]= getResultLabel(j);

}

selectWindow("Results");

run("Close");

open(dirArea48hrs+list48[i]);

for (j=0;j<nResults;j++){

Area48hrs[j]=getResult("Area",j);

}

selectWindow("Results");

run("Close");

for (j=0;j<Area6days.length;j++){

long=Area6days[j];

short=Area48hrs[j];

Arearatio[j]=long/short;

Arealogratio[j]=log(Arearatio[j])/log(10);

}

for (j=0;j<controlA1N5.length;j++){

pos = controlA1N5[j]-1;

totallog = totallog + Arealogratio[pos];

}

meanlog=totallog/controlA1N5.length;

for (j=0;j<Arealogratio.length;j++){

normArealogratio[j]=Arealogratio[j]/meanlog;

}

for (m=0; m<Arearatio.length; m++) {

setResult("Label",m,AreaLabel[m]);

setResult("Area 6days:48 hrs Ratio",m, Arearatio[m]);

setResult("log-Area 6days:48 hrs Ratio",m, Arealogratio[m]);

setResult("norm log-Area 6days:48 hrs Ratio",m, normArealogratio[m]);

}

saveAs("Results", dir2+AreaLabel[0]+"Chlorophyll Area-6daysto48hrs-Ratio.xls");

}

close();

*Macro #6a takes the spreadsheets generated by Macro #5a and compiles all the normalized YFP intensities generated for each plate into one excel sheet.*

dirYFPratio=getDirectory("Choose Directory with YFP Ratio Excel Sheets");

dir2=getDirectory("Choose output file for compiled ratios");

listYFP=getFileList(dirYFPratio);

Array.sort(listYFP);

setBatchMode(true);

mutstart = 0;

mutend = 336;

muttotal = mutend-mutstart;

mutLabelYFP = newArray(muttotal*listYFP.length);

normYFPrat = newArray(muttotal*listYFP.length);

k=0;

for (i=0; i<listYFP.length; i++) {

showProgress(i+1, listYFP.length);

open(dirYFPratio+listYFP[i]);

for(j=0;j<muttotal;j++){

normYFPrat[k] = getResult("log norm-Max: plus to no Vit YFP Ratio",j);

mutLabelYFP[k] = getResultLabel(j);

k=k+1;

}

}

selectWindow("Results");

run("Close");

for (i=0; i<normYFPrat.length; i++) {

setResult("Label",i,mutLabelYFP[i]);

setResult("norm log-Max: plus to no Vit YFP Ratio",i,normYFPrat[i]);

}

saveAs("Results", dir2+"cpUPR-mutants-compiled-YFPRatio.xls");

close();

*Macro #6b takes the spreadsheets generated by Macro #5b and compiles all the area ratio spreadsheets generated for each plate into one excel sheet.*

dirArea48hrs=getDirectory("Choose Directory with Area at 48 hrs Excel Sheets");

dirArea6days=getDirectory("Choose Directory with Area at 6 days Excel Sheets");

dir2=getDirectory("Choose output file for Area ratios");

list48=getFileList(dirArea48hrs);

list6d=getFileList(dirArea6days);

Array.sort(list48);

Array.sort(list6d);

setBatchMode(true);

mutstart = 0;

mutend = 336;

muttotal = mutend-mutstart;

k=0;

mutLabelArea = newArray(muttotal*list48.length/2);

logArearat = newArray(muttotal*list48.length/2);

for (i=0; i<list6d.length; i+=2) {

showProgress(i+1, list6d.length);

open(dirArea6days+list6d[i+1]);

Area48hrs = newArray(nResults);

Area6days = newArray(nResults);

AreaLabel = newArray(nResults);

Arearatio= newArray(nResults);

Arealogratio= newArray(nResults);

for (j=0;j<nResults;j++){

Area6days[j]=getResult("Area",j);

AreaLabel[j]= getResultLabel(j);

}

selectWindow("Results");

run("Close");

open(dirArea48hrs+list48[i+1]);

for (j=0;j<nResults;j++){

Area48hrs[j]=getResult("Area",j);

}

selectWindow("Results");

run("Close");

for (j=0;j<Area6days.length;j++){

long=Area6days[j];

short=Area48hrs[j];

Arearatio[j]=long/short;

Arealogratio[j]=log(Arearatio[j])/log(10);

}

for(j=0;j<muttotal;j++){

mutLabelArea[k] = AreaLabel[j];

logArearat[k] = Arealogratio[j];

k=k+1;

}

}

for (m=0; m<logArearat.length; m++) {

setResult("Label",m,mutLabelArea[m]);

setResult("log-Area 6days:48 hrs Ratio",m, logArearat[m]);

}

saveAs("Results", dir2+"Chlorophyll Area-6daysto48hrs-CompiledRatio.xls");

close();
